# Relationship between High Blood Pressure and Microalbuminuria in Children Aged 6–9 Years in a South African Population

**DOI:** 10.3390/children7090131

**Published:** 2020-09-07

**Authors:** Edna Ngoakoana Matjuda, Constance R. Sewani-Rusike, Samuel Nkeh Chungag Anye, Godwill Azeh Engwa, Benedicta Ngwechi Nkeh-Chungag

**Affiliations:** 1 Department of Human Biology, Faculty of Health Sciences, Walter Sisulu University PBX1, 5117 Mthatha, South Africa; ednan@gmail.com (E.N.M.); crusike1@wsu.ac.za (C.R.S.-R.); 2 MBCHB Programme, Faculty of Health Sciences, Walter Sisulu University PBX1, 5117 Mthatha, South Africa; samuelanye@gmail.com; 3 Department of Biological and Environmental Sciences, Faculty of Natural Sciences, Walter Sisulu University PBX1, 5117 Mthatha, South Africa; gengwa@wsu.ac.za

**Keywords:** children, blood pressure, microalbuminuria, cardiovascular risk factors, high blood pressure

## Abstract

Though the association between high blood pressure and microalbuminuria is well established in adults, there is a paucity of information on microalbuminuria in children. This study investigated the relationship between high blood pressure and microalbuminuria in 6–9-year-old children. A cross-sectional study, which included 306 primary school children of age 6–9 years old from urban areas (n = 154) and rural areas (n = 152) of the Eastern Cape Province of South Africa, was conducted. Participants’ anthropometric data were determined and systolic blood pressure (SBP), diastolic blood pressure (DBP) and heart rate (HR) were measured and converted to BP percentiles for age, sex and height. Creatinine and albumin concentrations were assayed in early morning midstream urine and the albumin to creatinine ratio (ACR) was calculated. There was a 42.8% prevalence of elevated blood pressure/high blood pressure (E-BP/H-BP) and a 10.1% prevalence of microalbuminuria. Among the 131 children with E-BP/H-BP, 17 had elevated ACR with a prevalence of 13.95%. SBP and HR increased with increasing range of ACR and, furthermore, SBP was significantly (*p* < 0.05) higher in children with moderately and severely increased ACR. SBP was associated with ACR and increased SBP predicted microalbuminuria (*R*^2^ = 0.42, adj *R*^2^ = 0.039, *B*: 0.120, *p* = < 0.05). In conclusion, microalbuminuria was present in 6–9-year-old South African children of African Ancestry and a weak association was observed with SBP in children.

## 1. Introduction

Hypertension is the leading cause of cardiovascular diseases (CVDs) and premature death worldwide. Though the prevalence of hypertension has decreased in high-income countries in the past four years, the prevalence of hypertension has increased in low- and middle-income countries (LMICs). It is estimated that approximately 1.04 billion (31.5%) of people in LMICs are hypertensive [1]. The increasing global prevalence of hypertension or high blood pressure in LMICs is a public health problem of concern [2]. Though hypertension was generally considered to be a health problem in adults, it is becoming prevalent in children. A recent meta-analysis study in children below 19 years of age showed that the pooled prevalence of hypertension was 4%, with approximately 10% for pre-hypertension. This study showed that childhood hypertension was on the rise, with a 75% increase from 2000 to 2015. More so, this prevalence of hypertension range from 4.32% in children aged 6 years to 3.28% in those below 19 years and was highest (7.89) in children aged 14 years [3]. There is evidence suggesting that essential hypertension in adulthood can be tracked back to the early decades of life, having its roots in childhood and adolescence [4]. In support of previous epidemiological evidence from longitudinal cohort studies across the world which have shown hypertension in childhood to track into adulthood [5,6], findings from South Africa have also shown hypertension in children to track into adolescence [7]. There is evidence of an increasing prevalence of hypertension in South African children [8,9]. Studies have shown a high prevalence of hypertension, ranging from 12 to 19% in South African children, especially in children of African ancestry [7,10]. South Africa is one of the countries undergoing rural to urban transition and populations in this transition, particularly the rural areas inhabited by South Africans of African ancestry, have been shown to experience an increase in overweight, obesity and blood pressure due to changes in diet and sedentary lifestyle [11]. Hypertension remains one of the leading CVD risk factors in both adults and children [12,13], with substantial evidence of its association with CVDs in adulthood [13]. Moreover, among the markers of CVD risk in adults, microalbuminuria, which is an early sign of both systemic vascular and renal disease [14,15], has been found to be an independent predictor of CVD [16].

Microalbuminuria is a condition defined by increased excretion of small quantities of albumin in urine, which may arise from renal damage leading to leakage of albumin through the glomerular filtration barrier [17] but it may also result from impaired vascular function [18]. Clinically, microalbuminuria can be determined by measuring urinary albumin excretion (UAE) rate [19]. However, UAE shows a wide range of variation in children [17] depending on pre-sample collection activity. Estimation of urine albumin concentration or albumin to creatinine ratio (ACR) has been considered as a simple alternative to UAE [19]. Moreover, ACR is superior to UAE and comparable to 24 h urine collections [20]. Thus, an early morning urine sample for the ACR provides a more sensitive estimate of microalbuminuria. Microalbuminuria can be defined as a UAE of 30 to 299 mg/24 h in adults, which also correlates with an ACR of ≥30 mg/g creatinine on a spot urine specimen or an ACR ≥3 mg/mmol [21]. Though ACR has been validated in several epidemiological studies in adults for the estimation of microalbuminuria [22,23], there is a relative paucity of such studies in children [24]. There is no specific definition for microalbuminuria in children due to the lack of sufficiently robust data in healthy children [25]. As such, ACR definition for adults has been adopted for children. This was supported by a large population study conducted with approximately 2000 healthy Chinese children that established an upper range of 14.7 mg/g for creatinine in boys and 19.8 mg/g for girls [26], suggesting that it is reasonable to use the adult definitions of microalbuminuria in paediatric patients. In another study, the mean ACR in normal children above 6 years of age seemed to fall between 8 and 10 mg/g (males: 7.5 mg/g; females 9.6 mg/g) [27].

The presence of microalbuminuria in adult patients with essential hypertension has also been confirmed. Though the mechanism on how hypertension leads to microalbuminuria remains controversial, is it thought to be as a result of vascular endothelial dysfunction or damage in the kidney by hypertension [17,28]. Further, it is suggested that elevated aortic pulse pressure due to increased blood volume load can increase blood flow into the kidney altering the renal hemodynamic and consequently damaging the renal microvasculature [29]. Damage to the glomerular endothelium leads to glomerular leakage, permitting large proteins in blood such as albumin to pass through the glomerulus. As a result of the potential consequence of hypertension on renal dysfunction, routine measurement of albuminuria in hypertensive patients has been recommended and anti-hypertensive treatment has been introduced for cases of microalbuminuria in hypertensive patients [30]. However, because of the paucity of information on microalbuminuria in children, findings on the association of hypertension and microalbuminuria in adults cannot be simply extrapolated to children. Moreover, though a few cross-sectional studies have shown microalbuminuria to be related to hypertension in children, the major challenge of these studies has been the small sample sizes. Although the prevalence of hypertension in children of African ancestry is on the rise in South African, there is limited information on the relationship between microalbuminuria and high blood pressure in this population. Thus, in this study, we seek to investigate the relationship between high blood pressure and microalbuminuria with a larger population of South African children of African ancestry.

## 2. Materials and Methods

### 2.1. Study Design and Population

This was a stratified cross-sectional study that randomly recruited primary school children of African ancestry aged 6–9 years old in Libode, Mthatha, and East London of the Eastern Cape Province of South Africa. This study took place in primary schools of rural settings in Libode and primary schools of urban settings in Mthatha and East London. The sample size of this study was calculated using the formula: n = z_1−a/2_^2^ p·(1 − p)/d^2^, where n = sample size, z_1−a/2_^2^ = confidence interval, p = estimate population size, and d = desired precision. The calculated sample size of this study was three hundred and three (303) children. Therefore, a minimum of 150 children were recruited from each site; that is, rural and urban areas.

### 2.2. Ethical Considerations

This study was conducted in accordance with the guidelines provided in the reviewed version of the 2008 Helsinki Declaration and also respected the local and national regulations of South Africa. Ethical approval was obtained from the Health Sciences Ethics Committee of Walter Sisulu University, South Africa, with Ref. No.: 112/2018. After careful explanation of the purpose and aim of this study, written informed consent was obtained from the parents and legal guardians of the children before enrolment into the study. This study adhered to the standards of reporting and was in accordance with the National Data Protection Acts as the identity of participants was kept confidential. There were no important changes to the study protocol after commencement.

### 2.3. Inclusion/Exclusion Criteria

Children aged 6–9 years who were free from any cardiovascular and renal diseases were recruited for this study. Ill and physically challenged children with any self-reported comorbidity or cardiovascular diseases were excluded from the study.

### 2.4. Data Collection and Biochemical Analysis

Anthropometric measurements were performed according to the International Standards for Anthropometric Assessments [31] on all the participants. Participants’ height was measured using a wall-mounted Harpenden stadiometer and recorded to the nearest 0.1 centimetres (cm). Weight was measured using a wireless Tanita weight scale (BC1000, Tanita Corporation, Tokyo, Japan) connected to a computer. Personal details of the children including sex, age and height were entered into the computer and the body mass index (BMI) for each participant was calculated from weight and height as weight/height^2^ (kg/m^2^). Blood pressure (BP) was measured using an automatic sphygmomanometer (Omron M500, HEM-7321-D, Omron Corporation, Kyoto, Japan) after the participants had rested for 10 min while seated with their back supported and feet uncrossed on the floor. The child’s right arm, placed at heart level, was fitted a cuff with a bladder length of 90% and a width of 40% arm circumference. Three BP readings, namely systolic blood pressure (SBP), diastolic blood pressure (DBP) and heart rate (HR), were taken at 2 min intervals. The average of the second and third BP readings was determined and converted to BP percentiles for sex, age and height and classified according to the American Academy of Paediatrics (AAP) 2017 guideline as normotensive: SBP and DBP < 90th percentile, elevated BP: SBP and/or DBP ≥ 90th < 95th percentile or high BP: SBP and/or DBP ≥ 95th percentile [32]. Mean arterial pressure (MAP) was calculated using the formula: MAP = (SBP + (2 × DBP))/3. Early morning midstream urine was collected from all participants in sterile tubes and used to quantify creatinine and albumin. Creatinine was measured using the Roche Cobas 6000 analyser based on the principle of the enzymatic method, whereby creatinine is converted by creatininase to creatine, which is further converted to hydrogen peroxide (H_2_O_2_). H_2_O_2_ reacts with chromophore in the presence of peroxidase to form a colour product measured at 546 nm. Albumin was determined using an enzyme-linked immunosorbent assay kit (Elabscience, Houston, TX, USA; Catalogue No.: ab108788). Assays were conducted according to the manufacturers’ protocols. The urinary albumin to creatinine ratio (ACR) was calculated and classified as normal ACR (mg/mmol) < 3 and ACR ≥ 3 was defined as microalbuminuria [33].

### 2.5. Statistical Analysis

Data were analysed using STATA MP Version 14.1. A Shapiro–Wilk test was used to check the data for normality and parametric tests were employed. Results were presented as the mean ±95% confidence interval (CI). Univariate analysis was used to compare mean differences of study parameters based on location and sex. Analysis of variance (ANOVA) was used to compare mean differences between the various categories of ACR for BP measures followed by least significant difference (LSD) Post Hoc test for between-group differences. Chi-square analysis was used to compare the proportion of high BP as well as microalbuminuria between urban and rural children based on gender. Pearson’s correlation and linear regression were used to evaluate the relationship between high blood pressure and increased ACR (microalbuminuria). A 95% confidence interval was used and a *p* ≤ 0.05 was considered significant.

## 3. Results

### 3.1. Baseline Characteristics of Study Participants

Three hundred and six (306) children were recruited for this study, which included 152 (49.8%) children from rural areas and 154 (50.2%) from urban areas. Among the 152 children in the rural areas, there were 83 (54.6%) girls and 69 (45.4%) boys, while among the 154 children in urban areas there were 88 (57.1%) girls and 66 (42.9%) boys. The study groups did not differ in age, weight, height and BMI though all haemodynamic parameters (SBP, DBP, HR and MAP) were different (*p* < 0.001). Further, urinary creatinine, albumin and ACR levels were significantly (*p* < 0.001) different in all the groups (Table 1).

### 3.2. Prevalence of Elevated/High Blood Pressure and Microalbuminuria

The prevalence of high blood pressure was 10.5% (32/306), while elevated/high blood pressure in the study population was 42.8% (129/306). High blood pressure as well as elevated blood pressure were more prevalent in rural than urban children and generally higher in girls than boys. The prevalence of microalbuminuria in the study population was 10.1% (31/306) and was more prevalent in female (7.2%) than male (2.9%) children. More so, the prevalence of microalbuminuria was significantly (*p* < 0.001) higher in rural (6.5%) than urban (3.6%) children. Among the 131 children with elevated or high blood pressure, 18 had microalbuminuria with a prevalence of 13.95% and it was greater in female than male children as well as in rural than in urban children. Results are summarized in Table 2.

### 3.3. Relationship between Blood Pressure Parameters and Albumin to Creatinine Ratio in Children

**Figure 1:**
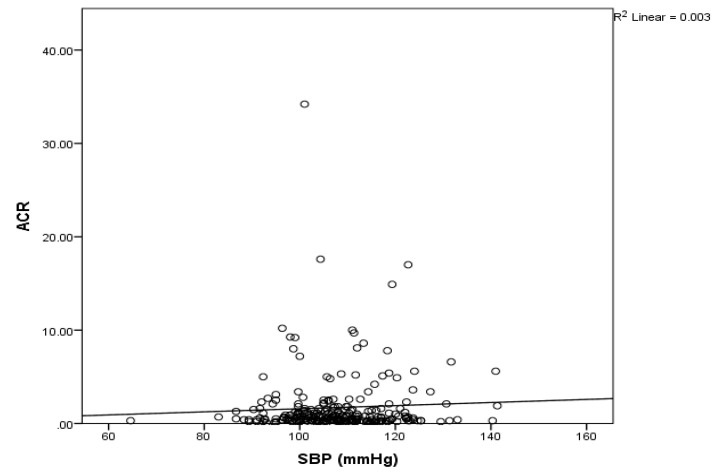
Correlation between systolic blood pressure (SBP) and albumin to creatinine ratio (ACR). There was a positive linear relationship with R^2^ =0.003.

Mean SBP and HR increased with increasing range of ACR in children. Children with microalbuminuria (i.e., ACR ≥ 3) had significantly higher (*p* < 0.05) mean SBP and HR compared to children who had ACR < 3. On the other hand, DBP did not show any specific pattern with the increasing range of ACR in children as there was no significant difference (*p* > 0.05) in the HR level between children with ACR < 3, ACR >3-30 and ACR > 30 (Table 3). Further, SBP was positively associated (correlation coefficient: *r* = 0.206; *p* < 0.05) with ACR level (Figure 1) and increased SBP was shown to predict microalbuminuria (coefficient of determination: *R*^2^ = 0.42, adj. *R*^2^ = 0.039, regression coefficient: *B*: 0.120, 95% CI: 0.048–0.192; *p* = 0.001).

## 4. Discussion

A possible relationship between high blood pressure and microalbuminuria was observed in this study. Hypertension is a global problem that is becoming more prevalent in children [34,35]. A recent meta-analysis study drawn from 47 articles showed a 4% prevalence of high blood pressure and 9.67% for elevated blood pressure in children [3]. Findings from this study showed a 10.5% prevalence of high blood pressure and a 32.3% prevalence of elevated blood pressure in South African children of African ancestry which was higher in female than male. This elevated blood pressure which was found among school-going children in this study is not a new phenomenon in Africa. A study conducted in Gambia found that the prevalence of high blood pressure in children aged 5–9 years old was 9.8% and the prevalence was higher in females [34]. Furthermore, a study conducted in urban and rural settings of Der es Salaam in Tanzania found that the prevalence of pre-hypertension/hypertension was 15.2% [35]. In this study, the prevalence of elevated or high blood pressure was higher in rural children compared to their urban counterparts. Further, SBP, DBP, and MAP were significantly higher in rural children compared to urban children. The increased prevalence of high blood pressure or hypertension may be attributed to rapid urbanization and lifestyle changes in rural areas in South Africa [11]. Since hypertension remains one of the leading risk factor of CVDs [36] and microalbuminuria is an independent risk factor of both cardiovascular and renal diseases [26], we sought to investigate the relationship between hypertension and microalbuminuria in children.

Microalbuminuria defined by abnormal or supranormal urinary excretion of albumin is a well-known independent predictor of CVD morbidity and mortality [37]. Although there have been large population-based studies on the prevalence of microalbuminuria in adults, there have been fewer studies in children. A 10–15% prevalence of microalbuminuria was observed in a study involving a general population of all age groups [38]. In the United States of America, a prevalence of 7.8% involving children, adolescents, and adults was reported by Jones and colleagues [39]. A study using NHANES 1999–2004 data found an 8.9% prevalence of microalbuminuria in adolescents aged 12–19 years [40]. Based on ACR ≥ 3 mg/mmol, the prevalence of microalbuminuria in this study was 10.1% and was higher in rural children than urban children. This finding suggests that renal dysfunction may be higher in rural than urban children. More so, microalbuminuria was higher in female than male children. This finding is consistent with previous findings which have shown females with higher microalbuminuria levels compared to males [41]. Factors such as female gender, puberty, weight and height, have also been associated with higher albumin excretion rate in healthy children [42].

The presence of microalbuminuria in adults with high blood pressure is well established and it is usually present as a marker for CVDs as well as impaired renal function [43]. Hypertension is suggested to cause renal impairment by damaging the glomerular endothelium and alter renal haemodynamic. Further, hypertension causes an increase in transforming growth factor-β1 and angiotensin II, obstructing the lysosomal degradation pathway which consequently leads to albuminuria [44]. It is generally accepted that SBP, and to a lesser extent pulse pressure, is the main determinant of albuminuria [45]. Previous studies have shown an association between microalbuminuria and high blood pressure in an adult population [46] as microalbuminuria was prevalent in adults with hypertension. Large population studies have shown a varying prevalence of microalbuminuria in hypertensive adults, ranging between 8% and 23% [47,48]. However, there are very few studies which have investigated the relationship between microalbuminuria and hypertension in children. A study showed an approximately 20% prevalence of microalbuminuria in children with primary hypertension [49], another showed a higher prevalence [50], while a study showed no prevalence of microalbuminuria in hypertensive children [16]. In this present study, a 13.95% prevalence of microalbuminuria was observed in children with elevated or high blood pressure. Further, a significantly increased SBP was observed in children with microalbuminuria. More so, there was a weak association between SBP and ACR and increased SBP which predicted microalbuminuria was observed implying that an increase in SBP will lead to microalbuminuria. These findings suggest that microalbuminuria was possibly associated with high blood pressure in children, perhaps indicating that high blood pressure in children may be implicated in the development of microalbuminuria. Possible mechanisms of the onset of microalbuminuria could be as a result of renal or vascular damage due to high blood pressure [27]. The presence of microalbuminuria in children with hypertension is of public health concern, as hypertension in adulthood, which is a major risk factor of CVD, tracks back to the early decades of life. A study has shown hypertension in childhood to be associated with microalbuminuria in adulthood in African Americans but not in Caucasians [51]. This suggests that African children who are closely related to African Americans may also be susceptible to blood pressure-related renal and vascular damage in adulthood. The European Society of Hypertension has suggested routine measurement of microalbuminuria as one of their recommendations in the management of hypertension in children though the assessment of microalbuminuria has not been fully established [52]. Though microalbuminuria has not been considered as a diagnostic marker in hypertensive South African children, these findings in this present study may be of concern for health policy consideration.

This study did not measure blood pressure in three different occasions as per the AAP 2017 guideline [32] to diagnose hypertension in children and therefore children with blood pressure above 95 percentile were classified as having high blood pressure rather than hypertension. Further, this study did not report the number of people that were excluded from this study. However, only participants that met the inclusion criteria and gave their consent were included for the study, and therefore, there was no bias in the recruitment of participants.

## 5. Conclusions

There was a weak association between systolic blood pressure and microalbuminuria in South African children of African ancestry. Although a low prevalence of microalbuminuria was observed, a high prevalence of high blood pressure was observed. The presence of microalbuminuria in children with high blood pressure calls for public health attention. Further studies are needed with more robust study design to confirm these findings. Further, long-term prospective studies in children should be conducted to properly diagnose hypertension and assess the potential cardiovascular and renal risk of microalbuminuria in hypertensive children.

## Figures and Tables

**Table 1:** Baseline characteristics of participating children.

	Rural (95% CI)		Urban (95% CI)		*p* Value
	Girls	Boys	Girls	Boys	
**N**	83	69	88	66	
**Age (yrs)**	7.91 (7.65–8.17)	7.88 (7.56–8.19)	8.34 (8.08–6.67)	8.11 (7.72–8.49)	0.320
**Height (m)**	1.25 (1.23–1.27)	1.27 (1.24–1.29)	1.29 (1.25–1.34)	1.28 (1.25–1.30)	0.198
**Weight (kg)**	25.44 (24.40–26.40)	27.46 (25.11–29.82)	28.61 (26.60–30.61)	28.10 (26.03–30.17)	0.137
**BMI (kg/m^2^)**	16.4 (15.8–16.9)	16.8 (15.8–17.9)	17.2 (16.4–18.0)	17.1 (16.0–18.2)	0.276
**SBP (mmHg)**	108.81 (106.05–111.57)	107.97 (104.02–111.91)	108.40 (104.98–111.81)	107.73 (104.72–110)	<0.001
**DBP (mmHg)**	71.1 (69.08–73.12)	68.43 (65.85–71.02)	69.30 (66.88–71.73)	69.52 (66.17–72.88)	<0.001
**HR (bpm)**	89.81 (86.32–93.30)	89.41 (86.34–92.48)	93.23 (90.42–96.03)	87.57 (84.37–90.78)	<0.001
**MAP (mmHg)**	83.67 (81.60–85.74)	82.18 (79.87–84.50)	82.33 (79.82–84.85)	82.54 (79.54–85.54)	<0.001
**Urine Creatinine (mmo/L)**	7.17 (6.16–8.11)	8.65 (6.52–10.78)	10.79 (9.03–12.57)	8.46 (6.53–10.37)	<0.001
**Urine Albumin (mg/L)**	47.06 (−7.48–101.71)	38.66 (−9.75–87.07)	41.78 (−9.09–92.65)	5.02 (2.65–7.38)	<0.001
**Urinary ACR (mg/mmol)**	6.16 (−0.01–12.33)	3.40 (−0.90–7.71)	4.17 (−1.51–9.85)	0.58 (0.38–0.77)	<0.001

Values are expressed as the mean (min CI–max CI); CI: confidence interval; N: number of children; Age (yrs): age in years; BMI: body mass index; SBP: systolic blood pressure; DBP: diastolic blood pressure; HR: heart rate; MAP: mean arterial pressure; ACR: albumin to creatinine ratio.

**Table 2:** High blood pressure and microalbuminuria among children.

Prevalence	Cohort N (%)			Rural N (%)		Urban N (%)	
	Cohort	Girls	Boys	Girls	Boys	Girls	Boys
NT	177 (57.8)	97 (31.7)	80 (26.1)	38 (12.4)	43 (14.1)	59 (19.3)	37 (12.1)
E-BP	99 (32.3)	53 (17.3)	44 (14.3)	33 (10.8)	20 (6.5)	20 (6.6)	24 (7.9)
H-BP	32 (10.5)	21 (6.9)	11 (3.6)	12 (3.9)	6 (2.0)	9 (2.9)	5 (1.6)
E-BP /H-BP	131 (42.8)	74 (24.2)	55 (17.9)	45 (14.7)	26 (8.5)	29 (9.5)	29 (9.5)
NAU	275 (89.9)	149 (48.7)	126 (41.2)	71 (23.2)	61 (19.9)	78 (25.5)	65 (21.2)
MAU *	31 (10.1)	22 (7.2)	9 (2.9)	12 (3.9)	8 (2.6)	10 (3.3)	1 (0.3)
MAU in E-BP/H-BP	18(13.95)	13 (10.85)	4 (3.1)	9 (7.0)	4 (3.1)	3 (2.3)	1 (0.4)

N: number of children; NT: normotension; H-BP: high blood pressure; E-BP: elevated blood pressure; NAU: normal albuminuria; MAU: microalbuminuria; NAU: ACR < 3; MAU: ACR ≥ 3, * indicates significant difference between locations based on gender.

**Table 3:** Effect of haemodynamic parameters on albumin to creatinine ratio ranges.

ACR Range	<3	3–30	>30	*p* Value
SBP Mean (95% CI)	107.2 (105.7–108.6)	112.3 (107.4–116.6)	143.1 (56.8–229.5) ^a^	<0.01
DBP Mean (95% CI)	68.9 (67.6–69.9)	71.8 (67.3–76.2)	69.3 (63.7–74.9)	>0.05
HR Mean (95% CI)	90.3 (88.1–91.9)	90.8 (85.3–96.5)	91.1 (82.6–90.6)	>0.05

SBP: systolic blood pressure; DBP: diastolic blood pressure; HR: heart rate; ACR = albumin to creatinine ratio; ACR < 3: slightly increased ACR; ACR > 3–30: moderately increased ACR; ACR > 30: severely increased ACR; ^a^ indicates significant difference with <3 ACR range; CI: confidence interval.
